# Developing *Penicillium digitatum* Management Strategies on Post-Harvest Citrus Fruits with Metabolic Components and Colonization of *Bacillus subtilis* L1-21

**DOI:** 10.3390/jof8010080

**Published:** 2022-01-14

**Authors:** Yongmei Li, Mengyuan Xia, Pengbo He, Qiaoming Yang, Yixin Wu, Pengfei He, Ayesha Ahmed, Xiangsong Li, Yuehu Wang, Shahzad Munir, Yueqiu He

**Affiliations:** 1 State Key Laboratory for Conservation and Utilization of Bio-Resources in Yunnan, Yunnan Agricultural University, Kunming 650201, China; kala.111@163.com (Y.L.); xiamengyuan@mail.kib.cn (M.X.); pengbohe@126.com (P.H.); Yang2021090930@163.com (Q.Y.); WY68579@126.com (Y.W.); nanhudaozhu@163.com (P.H.); aisha_ahmed01@hotmail.com (A.A.); lixiangsongyn@163.com (X.L.); 2 Yunnan Key Laboratory for Fungal Diversity and Green Development, Kunming Institute of Botany, Chinese Academy of Sciences, Kunming 650201, China; wangyuehu@mail.kib.ac.cn; 3 Faculty of Agronomy and Biotechnology, Yunnan Agricultural University, Kunming 650201, China

**Keywords:** fungi, pathogen, *Penicillium digitatum*, *Bacillus subtilis* L1-21, LC-MS

## Abstract

Citrus is among the most important plants in the fruit industry severely infected with pathogens. Citrus green mold caused by *Penicillium digitatum* is one of the most devastating diseases during post-harvest stages of citrus fruit. In this study, a potential endophyte *Bacillus subtilis* L1-21, isolated from healthy citrus plants, was assessed for its biocontrol activity against the pathogen *P. digitatum*. Based on an in vitro crosstalk assay, we suggested that *B. subtilis* L1-21 inhibits the pathogen with an inhibition zone of 3.51 ± 0.08 cm. Biocontrol efficacy was highest for the fermented culture filtrate of *B. subtilis* L1-21. Additionally, using GC-MS analysis, 13 compounds were detected in the extract of this endophyte. The culture filtrate in Landy medium could enlarge and deform pathogen spores and prevent them from developing into normal mycelium. Accordingly, the Landy culture filtrate of *B. subtilis* L1-21 was stable in the temperature range of 4–90 °C and pH of 3–11. Further, MALDI-TOF-MS for *B. subtilis* L1-21 detected surfactin, fengycin, bacillaene and bacilysin as potential antifungal compounds. GFP-tagged *B. subtilis* L1-21 easily colonized in citrus fruit peel and pulp, suggesting its role in eliminating the fungal pathogen. Altogether, it is highly expected that the production of antifungal compounds, and the colonization potential of *B. subtilis* L1-21 are required against the post-harvest *P. digitatum* pathogen on citrus fruit.

## 1. Introduction

Citrus is a general term for citrus fruit trees, mainly including pomelo, mandarin, tangerine, lemon, sweet orange, grapefruit and lime [1]. The planting and yield of citrus fruit rank first in the fruit industry and give an idea of the importance of this fruit in international agricultural trade [2]. Citrus fruit is easily infected with various pathogens during postharvest storage and transportation, resulting in decay and deterioration, and economic losses, leading to adverse effects on the development of the citrus industry [3,4]. Postharvest disease in citrus fruit caused by *Penicillium* is one of the most severe diseases with losses of at least 10% and, in severe cases, up to 90% [5,6]. Among them, the losses caused by the green mold of *P. digitatum* is the most devastating. The necrotic fungus gains entry in the fruit tissues through wounds and has a short life cycle of 3 to 5 days but produces a profound number of conidial spores that could initiate a new life cycle [7], spreading the infection exponentially. Thus, effective control measures are very important to avoid further loss.

Traditional practices widely used for the management of *Penicillium* largely depend upon chemical fungicides such as pyrimethanil, imazalil, thiaben-dazole, prochloraz or different mixed formulations of these compounds [8]. However, the repetitive and excessive use of the same fungicides pose a threat to human and environmental health as well as the development of resistance in pathogensmake disease control near to impossible [9]. Thus, an effective and ecologically friendly method for post-harvest disease control is imperative. In this regard, potential bioagents have been reported for controlling citrus green mold and blue mold caused by *Penicillium* [10]. Additionally, the endophyte-medicated biocontrol of pathogens offers enhanced green agriculture and safe means for disease control, thus avoiding the adverse effects of chemical control [11,12]. It also showed positive effects on postharvest diseases and significantly maintained the fruit quality as well [13]. As it is generally regarded as safe (GRAS), biocontrol agents such as *Bacillus* spp. have been widely studied and reported for many years to manage plant diseases and improve plant survival [13,14,15]. In particular, *B. amyloliquefaciens*, *B. subtilis*, *B. velezensis*, *B. megaterium*, *B. pumilus* and *B. cereus* have effective antagonistic activity against a variety of plant pathogens, including fungi, bacteria and oomycetes [16,17]. Potential *Bacillus* spp. could control green mold caused by *P. digitatum* [18,19]. Other microorganisms for controlling citrus fruit green mold include *Pseudomonas* [20], *Paenibacillus* [21] and *Aureobasidiuma* [22].

*Bacillus* could control green mold through different interesting mechanisms and strategies. These mechanisms include induced resistance, wound colonization, competition with a pathogen for sites and nutrients, antifungal compounds, inhibition of spore germination, deformation of mycelia and disruption of the cell wall [19,23,24]. Numerous lipopeptide compounds from potential bacterial strains have been isolated, purified and identified, such as surfactin, fengycin and iturin [25,26], macrolactin and bacillaene. These compounds are known to inhibit *P. digitatum* [24].

In our previous study, we reported that endophyte *B. subtilis* L1-21 regulates important metabolites in huanglongbing (HLB)-affected citrus plants [14]. However, the role of this endophyte was unknown in the citrus fruit during storage. Using the candidate citrus endophytic microbiome during citrus fruits storage could help in reducing the pathogen incidence and severe losses to the citrus industry. Therefore, the present study was aimed at isolating and identifying potential pathogens of citrus green mold, and to improve the biocontrol efficacy of endophyte *B. subtilis* L1-21. The colonization potential of this endophyte was revealed in detail during the pathogen interaction inside the citrus fruit. Furthermore, we elucidated the mode of action in conjunction with metabolic compounds produced by *B. subtilis* L1-21 to develop an efficient strategy for protection against citrus fruit green mold diseases.

## 2. Materials and Methods

### 2.1. Bacterial Strains and Culture Conditions

*Bacillus subtilis* L1-21 was previously isolated from healthy citrus plant [14]. The endophytic strain was stored at −80 °C in 50% glycerol stock, and the pure culture was inoculated into nutrient broth (NB) for further use. The culture filtrate of *B. subtilis* L1-21 was prepared in Luria Bertani (LB) medium at 30 °C at 160 rpm for 16 h, and 1% (*v*:*v*) was inoculated in the above medium at 160 rpm at 30 °C. Samples were taken every day until the 4th day. After fermentation, the medium was centrifuged at 10,000 rpm for 15 min and then the supernatant was filtrated using 0.22 μm filter paper. The green fluorescence protein (GFP) labelled *B. subtilis* L1-21 was obtained through transforming the vector pYC127 [27], hence named as *B. subtilis* L1-21-GFP.

### 2.2. Fruit Materials

Mandarin (*Citrus reticulata* cv. Shatangju) fruits were harvested from an orchard located in Guiling Guanxi Province, China. The citrus plants present in the orchard were not given any chemical treatment. The fruit brought to the laboratory were rinsed for 3 min with flowing water followed by surface sterilization with 2.5% of sodium hypochlorite for 2 min, washed with distilled water, 75% of ethanol (*v*/*v*) for 1 min, washed three times with sterile distilled water, and air-dried at room temperature in laminar flow hood for further use. 

### 2.3. Isolation and Identification of Potential Penicillium Isolates

The conidia from the diseased citrus fruit were picked with a sterilized wire loop on an ultra-clean workbench and cultured on potato dextrose agar (PDA) medium at 25 ± 1 °C for 3 d. After purification, the conidia were stored at 4 °C for a short period. In order to check the pathogenicity of isolate, spores were inoculated at the equator of citrus fruit with concentrations of 10^3^ spores mL^−1^, 10^4^ spores mL^−1^ and 10^5^ spores mL^−1^, and kept at 25 ± 1 °C. The citrus fruits were observed for pathogenicity after 4 d. Further, for molecular identification of the pathogenic fungus, the fungus stored at 4 °C was inoculated on PDA medium at 25 ± 1 °C with a sterilized wire loop on an ultra-clean workbench and cultured for 3 d at 25 ± 1 °C for DNA extraction. After that, the mycelia and spores were harvested, the slightly modified cetyl trimethylammonium bromide (CTAB) method was used to extract genomic DNA [28]. ITS1 (TCCGTAGGTGAACCTGCGG) and ITS4 (TCCTCCGCTTATTGATATGC) were used for identification. The PCR reaction conditions were as follows: pre-denaturation at 94 °C for 4.5 min; 30 cycles of 94 °C denaturation for 30 s, 56 °C annealing for 30 s, 72 °C extension for 45 s, followed by a 10-min extension at 72 °C. PCR products of targeted bands were sent to Kunming Qingke Biotechnology Co., LTD, China for sequencing. The obtained sequences were trimmed and submitted to the National Center for Biotechnology Information (NCBI). BLAST was performed to check for a homologous search against microbial genomes. In total, 9 sequences from the matched results were used to construct the phylogenetic tree of strain L1 with the software MEGA X.

### 2.4. Cross-Talk Experiment between Fungal Pathogen and Potential Endophyte

Culture of *B. subtilis* L1-21 was obtained on NB agar after 24 h. Petri dishes (9 cm) containing 15 mL of PDA were prepared. In total, 100 μL of pathogen suspension (1.0 × 10^5^ spores mL^−1^) was applied to the PDA medium and kept for few minutes for drying [13]. *B. subtilis* L1-21 was inoculated at four symmetrical points 2.5 cm from the center for 2 d at 25 ± 1 °C. After 2 d, the diameter of the inhibition zone was measured accordingly.

### 2.5. Biocontrol Efficacy Assay

One puncture was made with a sterile tip at a 1-cm depth at the equator of citrus fruit. *B. subtilis* L1-21 cells and *P. digitatum* spores were suspended in a 0.85% sodium chloride solution. The punctures were inoculated with the following treatments: (i) control 1, 10 μL of *P. digitatum* spore suspension (1 × 10^4^ spores mL^−1^) and 10 μL of 0.85% sodium chloride solution; control 2, 10 μL of *P. digitatum* spore suspension (1 × 10^4^ spores mL^−1^) and 10 μL of NB culture; (ii) 10 μL of *P. digitatum* spore suspension 1 × 10^4^ spores mL^−1^) and 10 μL of *B. subtilis* L1-21 cell suspension (without culture filtrate, 1 × 10^8^ CFU mL^−1^); (iii) 10 μL of *P. digitatum* spore suspension and 10 μL of *B. subtilis* L1-21 culture filtrate; and (iv) 10 μL of *P. digitatum* spore suspension and 10 μL of *B. subtilis* L1-21 fermentation (cell and culture filtrate). Control 1 was set for treatment (ii) and control 2 was set for treatment (iii) and (iv). Each replication consisted of 30 citrus fruits and each treatment consisted of three replications. The modified disease fruit grading standard was as following [29]: Grade 0, healthy; Grade 1, mold area ≤ 20%; Grade 2, mold area >20 and ≤ 40%; Grade 3, mold area >40 and ≤ 60%; Grade 4, mold area > 60 and ≤ 80%; Grade 5, mold area > 80%.

(1) $$\mathrm{Disease}\;\mathrm{index}=\frac{\sum (\;\mathrm{Number}\;\mathrm{of}\;\mathrm{disease}\;\mathrm{of}\;\mathrm{fruit}\times \;\mathrm{number}\;\mathrm{of}\;\mathrm{Grade}\;)}{\mathrm{Total}\;\mathrm{number}\;\mathrm{of}\;\mathrm{fruit}\times \;\mathrm{highest}\;\mathrm{number}\;\mathrm{of}\;\mathrm{Grade}}$$

(2) $$\mathrm{Biocontrol}\;\mathrm{efficacy}=\frac{\;\mathrm{Disease}\;\mathrm{index}\;\mathrm{of}\;\mathrm{control}-\;\mathrm{disease}\;\mathrm{index}\;\mathrm{of}\;\mathrm{treatment}}{\;\mathrm{Disease}\;\mathrm{index}\;\mathrm{of}\;\mathrm{control}}\times 100\%$$

### 2.6. Identification of Bacillus Subtilis L1-21 Extracts Using GC-MS

In order to prove how this *B. subtilis* L1-21 displayed a marked inhibitory effect against the pathogen, *B. subtilis* L1-21 was cultured in NB medium and incubated at 30 °C and 160 rpm for 4 d. The cells were removed after centrifugation, 200 mL of supernatant was extracted with 200 mL of ethyl acetate 3 times, 600 mL of ethyl acetate was evaporated by rotation, and cells were finally dissolved in 4 mL of ethyl acetate. GC–MS analysis was operated using a gas chromatography (7890-5975c, Agilent, United States) equipped with DB-5MS (30 m × 0.25 mm × 0.25 μm) and a mass spectrometer (EI with replaceable horn). The temperature program was set at an initial temperature of 40 °C for 1 min, and then increased to 130 °C at 10 °C min^− 1^, maintained for 5 min and then increased to 230 °C at 8 °C min^−1^, and maintained for 5 min; in total, 30.5 min. High-purity helium was used at a constant flow rate of 1 mL min^−1^. The mass spectrometer was equipped with a 70 eV electron impact ionization (interface temperature of 280 °C, ion source temperature of 230 °C) and a quadrupole analyzer. The ions were detected in the range of 50–600 *m*/*z*. Finally, results of mass spectra were analyzed using Chemistation software (Agilent, United States).

### 2.7. Screening Medium for Producing Antifungal Substances

NB medium, MT medium (peptone, 1 g L^−1^; glucose, 1 g L^−1^; CaCl_2_, 1 g L^−1^; K_2_HPO_4_, 1 g L^−1^; MgSO_4_, 0.2 g L^−1^; FeCl_3_, 0.01 g L^−1^; pH = 7.0), R_2_A medium, tryptic soya gar (TSA) medium, 10% TSA medium and Landy medium were selected for screening. The culture filtrate of *B. subtilis* L1-21 was prepared in LB medium at 30 °C and 160 g for 16 h, and 1% (*v*:*v*) was inoculated in the above medium at 160 rpm and 30 °C. Samples were taken every day until the 4th d. After fermentation, the medium was centrifuged at 10,000 rpm for 15 min and then the supernatant was filtrated through 0.22 μm filter paper. After mixing (1:1, *v*/*v*) culture filtrate with PDA (filtration) containing 0.8% (*w*:*v*) agar, 50 μL of the mixture was put onto the sterilized glass slide; 40 μL of spore suspension (10^6^ spores mL^−1^) was added to the solidification drop, and was incubated at 25 ± 1 °C; the spore germination was observed and counted after 32 h. The medium without inoculating *B. subtilis* L1-21 was used as the control. Prochloraz was used as positive control with a concentration of 450 mg/L (Zhejiang Tianfeng Biological Science Co., Ltd. China). The spore germination was observed under microscope (ZEISS) and the spore germination rate was calculated. More than 200 spores were counted for each treatment and three replications were used for each. The morphology of spores was observed using scanning electron microscope (ZEISS Sigma 300, Germany; Turbo Freeze Dryer with Turbo Freeze Dryer with liquid nitrogen, Quorum, UK; Kunming Institute of Botany, Chinese Academy of Science).

(3) $$\mathrm{Germination}\;\mathrm{rate}=\frac{\;\mathrm{Number}\;\mathrm{of}\;\mathrm{germination}\;\mathrm{spore}}{\;\mathrm{Total}\;\mathrm{number}\;\mathrm{of}\;\mathrm{spore}}$$

(4) $$\mathrm{Inhibition}\;\mathrm{rate}=\frac{\;\mathrm{Germination}\;\mathrm{rate}\;\mathrm{of}\;\mathrm{control}\;-\;\mathrm{germination}\;\mathrm{rate}\;\mathrm{of}\;\mathrm{treatment}}{\;\mathrm{Germination}\;\mathrm{rate}\;\mathrm{of}\;\mathrm{control}}$$

### 2.8. Antifungal Stability of Bacillus Subtilis L1-21 Culture Filtrate

Temperature tolerance of *B. subtilis* L1-21 culture filtrate was checked using 500 μL of filtrate in the range of 50, 60, 70, 80 and 90 °C in a water bath for 30 min. Autoclave was used at 100 and 121 °C for 30 min and culture was brought to room temperature for bioassay experiments. Culture filtrate was kept at 4 °C for 7 d for a low temperature tolerance bioassay. Further, UV light treatment was given by exposing 500 μL of culture filtrate in a Petri dish (6 cm, without cover) to 365-nanometer UV light for 15, 30, 45, 60 and 121 min at a distance of 30 cm, and the experiment was repeated three times. In addition, L1-21 culture filtrate was adjusted to pH 1–12 using 0.5 M HCl and NaOH and placed at room temperature for 24 h, followed by an adjustment of pH 7 for bioassay, and the pH 6 served as the control group. Each treatment consisted of three replicates. After all the above treatments, effects of culture filtrate on spore germination rate of *P. digitatum* were determined to evaluate the stability of culture filtrate.

### 2.9. Characterization of the Antifungal Compounds Using LC-MS

The antifungal compounds were detected using LC-MS. Culture filtrate of *B. subtilis* L1-21 grown in 200 mL of Landy was dried at 50 °C, followed by an addition of 4 mL of methanol to dissolve the compounds. Chromatographic separation was carried out using a column (AQC18 column, 5.0 μm 4.6 × 250 mm, 5 μm, Welch Ultimate, China) with a flow rate of 1 mL min^−1^. A mobile phase consisting of 0.5% (*v*/*v*) trifluoroacetic acid (TFA) (A) and methanol (B) was used. The following linear gradient elution was used: 5% methanol at 0 min, increased to 100%, kept at 100% methanol from 60 to 70 min. The UFLC-MS-IT-TOF apparatus and method were used as performed previously by Li et al. [30]. The ions were detected in the range of 1–1000 *m*/*z* and 800–2000 *m*/*z*, respectively.

### 2.10. Colonization of GFP-Tagged Endophytic Strain

GFP-tagged *B. subtilis* L1-21 was put on shaking in NB medium (with chloramphenicol 10 μg mL^−1^) at 160 rpm with 37 °C for 2 d. The fruit were soaked in GFP-L1-21 (1 × 10^8^ CFU mL^−1^) for 30 min and kept at 25 ± 1 °C. Samples were taken at 1, 3, 5 and 7 d with three replicates in each treatment. After surface sterilization, 1 g of the fruit peel and pulp was ground and 9 mL of sterilized 0.85% (*w*/*v*) sodium hypochlorite was added. The 100-μL tissue solution was inoculated on a chloramphenicol (10 μg mL^−1^) NB plate and incubated at 37 °C for 24 h; the number of GFP colonies was counted. The colonization ability was further validated using a scanning electron microscope. In this experiment, one puncture was made with a sterile tip at a 1 cm depth at the equator of citrus fruit. The experiment was carried out in the following order: Control (CK) for 12 and 24 h; 10 μL of *B. subtilis* L1-21 cell suspension (without culture filtrate, 1 × 10^8^ CFU mL^−1^) for 12 and 24 h; 10 μL of *P. digitatum* spore suspension for 24 h; 10 μL of *P. digitatum* spore suspension (1 × 10^4^ spores mL^−1^) and 10 μL of *B. subtilis* L1-21 cell suspension for 12 and 24 h. The fruits were kept at 25 ± 1 °C. The wound of citrus fruit was cut and then observed using a scanning electron microscope at 12 and 24 h.

### 2.11. Data Analysis 

All data were statistically analyzed using SPSS version 22 (SPSS Inc., Chicago, IL, USA) using a one-way analysis of variance (ANOVA). Duncan’s multiple range test was applied to determine the significant difference at *p* < 0.05. All data acquired from the repeated experiments were expressed using the mean ± standard deviation. 

## 3. Results

### 3.1. Morphological and Molecular Identification

**Figure 1:**
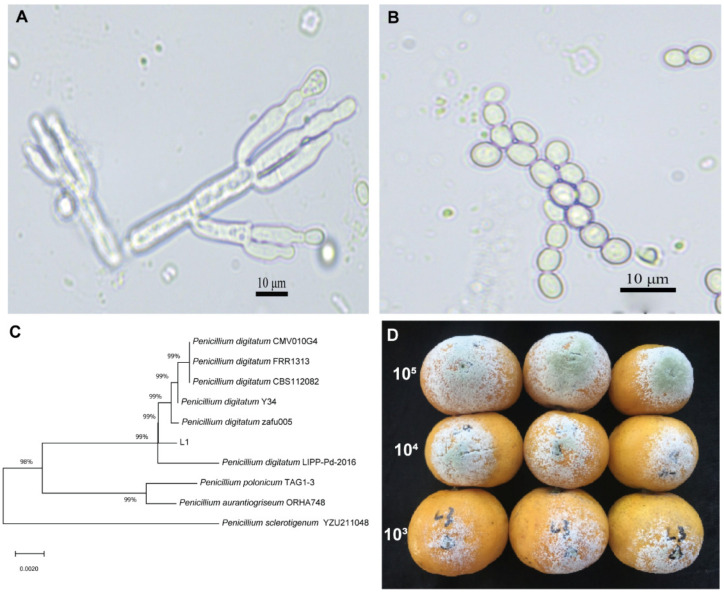
(**A**,**B**) Morphological characteristics of *Penicillium digitatum* strain L1 with conidiophores and spores. (**C**) Molecular phylogenetic tree of partial *18S rRNA* gene sequence of *P. digitatum* L1. (**D**) Pathogenicity of *P. digitatum* L1 spores at different concentrations on citrus fruits.

The macro and microscopic observations of the fungal isolate from citrus fruit confirmed that it belongs to genus *Penicillium* with depictions of conidiophores and spores (Figure 1A,B). The partial *18S rRNA* gene sequence was deposited to NCBI and a GenBank accession number was assigned (MZ881937). The results of the identity analysis of the sequences confirmed that they belonged to *Penicillium digitatum* (Figure 1C). *P. digitatum* L1 10^3^, 10^4^ and 10^5^ spores mL^−1^ upon inoculation into healthy citrus fruit caused fruit rot and green mold (Figure 1D).

### 3.2. In Vitro Biocontrol Efficacy and Effects of Bacillus Subtilis L1-21 on Penicillium Digitatum

**Figure 2:**
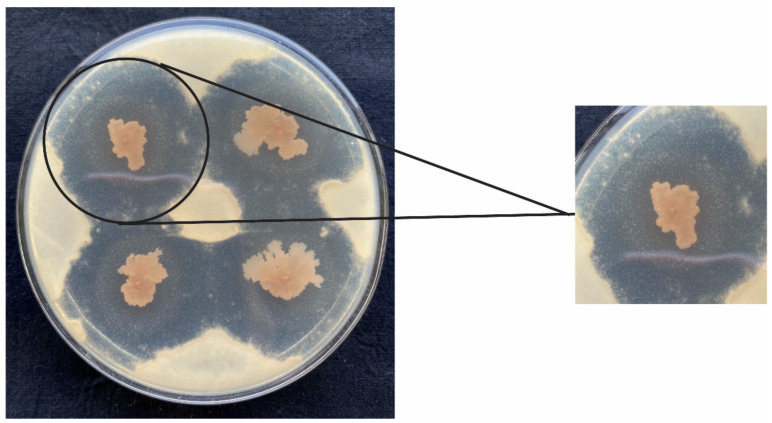
In vitro crosstalk assay between pathogen and *Bacillus subtilis* L1-21. The hollow zones around pink colonies display growth inhibition of pathogen by endophyte L1-21.

**Figure 3:**
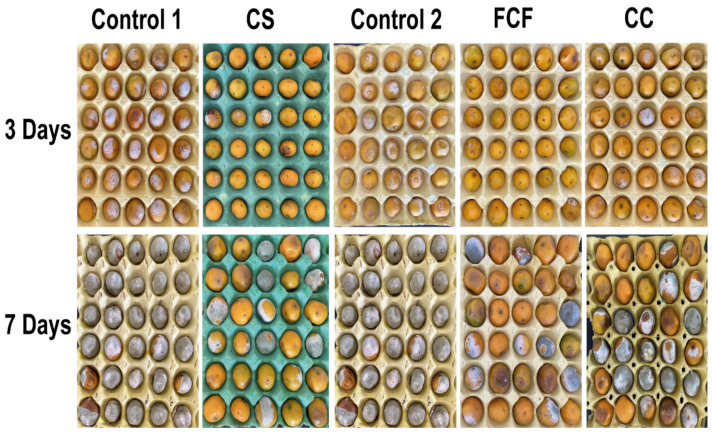
Biocontrol efficacy after 3 and 7 d have a significant inhibition of pathogens on citrus fruit. The treatments show a marked difference compared with the control, where the control effect is better at 3 d. CS, cell suspension; FCF, fermented cell filtrate; CC, culture filtrate; Control 1, water; Control 2, NB media.

**Figure 4:**
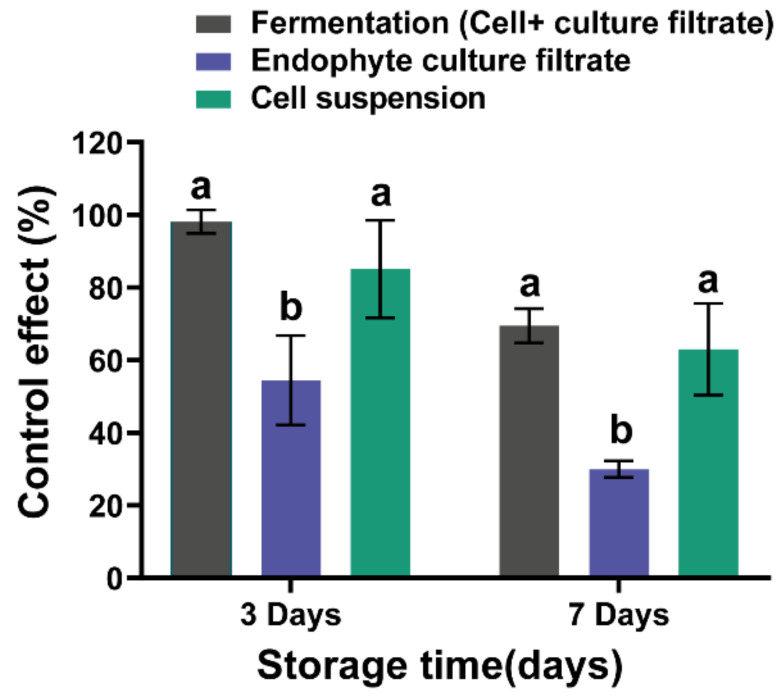
In vivo effects of different treatments of *Bacillus subtilis* L1-21 on citrus fruit. Different letters indicated a significant difference within the same time point according to the Duncan’s multiple range test (*p* < 0.05).

*Bacillus subtilis* L1-21 showed a potential inhibition effect on the mycelial growth of *P. digitatum* during co-culture assays on PDA (Figure 2). The diameter of each inhibition zone was observed as 3.51 ± 0.08 cm. After inoculating *P. digitatum* for 3 d in PDA medium, the biocontrol efficacies of fermentation in NB medium (cell and culture filtrate) treatment, cell suspension and culture filtrate treatment were 98.15 ± 3.21%, 54.47 ± 12.33% and 85.10 ± 13.45%, respectively (Figure 3 and Figure 4). After 7 d, the biocontrol efficacy of the fermentation treatment (cell and culture filtrate) of *B. subtilis* L1-21 appeared to be the highest, followed by the cell suspension of *B. subtilis* L1-21, and the culture filtrate treatment was recorded as the lowest. The results suggested that the biocontrol efficacy of all three treatments decreased over time. 

### 3.3. Extraction of Antifungal Compounds of Bacillus Subtilis L1-21 Using GC-MS

**Figure 5:**
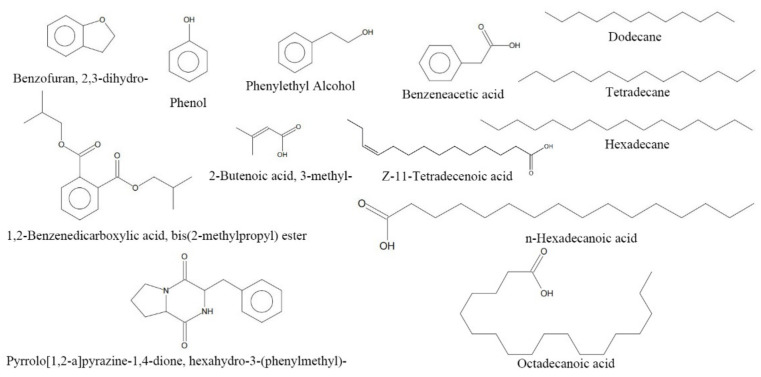
Chemical structures of the identified compounds from ethyl acetate extracts of *Bacillus subtilis* L1-21 using GC–MS.

A total of 44 peaks were detected using GC-MS, when the purified extract from supernatant of NB medium filtrate was loaded into the GC-MS for analysis (Figure S1). We identified 13 compounds (Figure 5) with a quality match of 85% minimum (Table 1), including 2-butenoic acid, 3-methyl-, phenol, phenylethyl alcohol, dodecane, benzofuran, 2,3-dihydro-, benzeneacetic acid, tetradecane, hexadecane, Z-11-tetradecenoic acid, pyrrolo[1,2-a]pyrazine-1,4-dione, hexahydro-3-(phenylmethyl)-, 1,2-benzenedicarboxylic acid, bis(2-methylpropyl) ester, n-Hexadecanoic acid and octadecanoic acid. Among them, the highest relative content was pyrrolo[1,2-a]pyrazine-1,4-dione, hexahydro-3-(phenylmethyl)-.

### 3.4. Culture Filtrate of Endophyte Bacillus Subtilis L1-21 on Spore Germination

**Figure 6:**
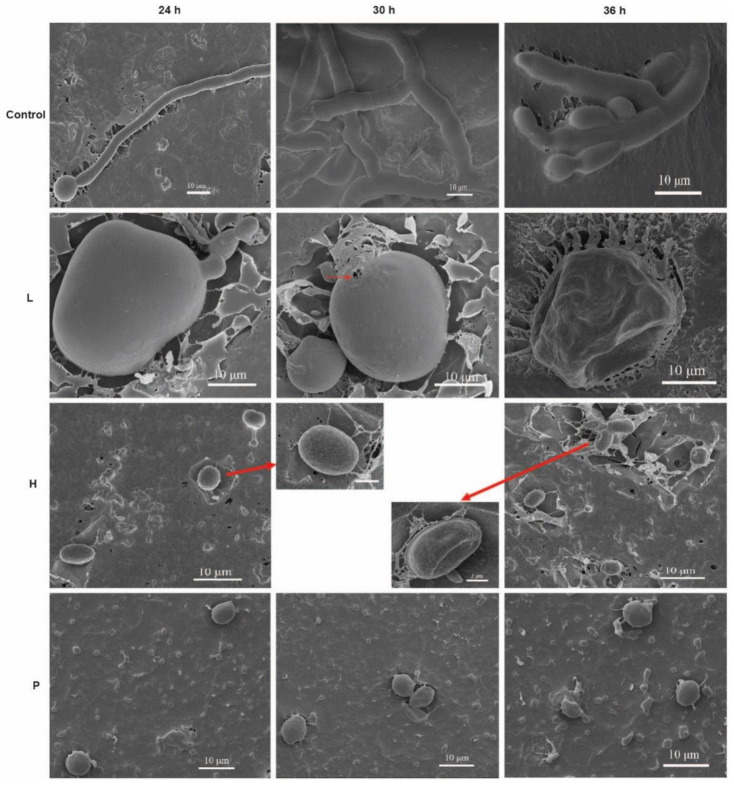
*Bacillus subtilis* L1-21 culture filtrate were tested against the pathogen. Spore germination and morphology were observed under a scanning electron microscope. Deformed spores were present in the presence of culture filtrate of *B. subtilis* L1-21. L means low concentration (5-fold dilution from higher concentration) of *B. subtilis* L1-21 Landy culture filtrate, H means high concentration of *B. subtilis* L1-21 Landy culture filtrate (*B. subtilis* L1-21 was cultured in Landy medium for 72 h), P means prochloraz (Fungicide widely used in China for controlling citrus fruit green mold) (positive control).

Six culture filtrates of *B. subtilis* L1-21 were tested to assess the difference in inhibition activity on spore germination. However, the highest inhibition was recorded for the culture filtrate of Landy medium (Table 2), but there were differences in the spore morphology. The low concentration of Landy culture filtrate could enlarge and deform spores; even with a small amount of spore germination, spores could not develop into mycelia normally (Figure 6). The high concentration of *B. subtilis* L1-21 culture filtrate in Landy medium could inhibit spore germination with the morphology of spores similar to the treatment of fungicide prochloraz for 72 h (Figure 6).

### 3.5. Stability of Bacillus Subtilis L1-21 Culture Filtrate under Different Conditions

**Figure 7:**
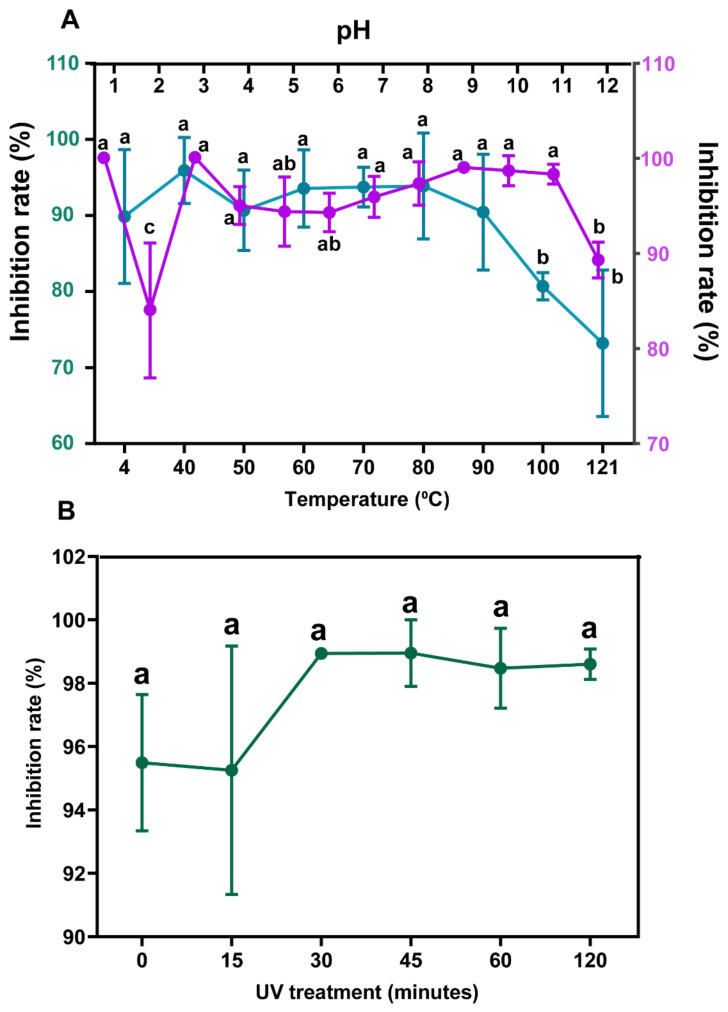
(**A**) Stability of *Bacillus subtilis* L1-21 in the presence of different temperatures and pH treatments. (**B**) UV effect on *B. subtilis* L1-21. Different letters on top indicate the significant differences (*p* < 0.05) over increasing temperature, pH and time.

The stability of *B. subtilis* L1-21 culture filtrate present in Landy medium was checked in the presence of different temperature ranges. No significant difference was detected ranging from 40–90 °C, but the inhibition rate of the endophyte was reduced at a temperature of 100 or 121 °C. Treatment for 30 min at 121 °C decreased the inhibition rate to 73.20% (Figure 7A). Except for pH treatments 2 and 12, the antifungal activity of the *B. subtilis* L1-21 culture filtrate could be kept or under strong-acid or strong-base treatment. The pH 2 treatment resulted in a decrease inhibition rate to 83.63%. Additionally, there was no significant difference among the different times of UV radiation from 15 to 120 min (Figure 7B). The effect of temperature on the antifungal activity of the *B. subtilis* L1-21 culture filtrate was stronger than that of pH or UV treatment when inhibition rates are concerned.

### 3.6. Characterization of Antifungal Compounds from Endophyte Bacillus Subtilis L1-21 Using LC-MS

**Figure 8:**
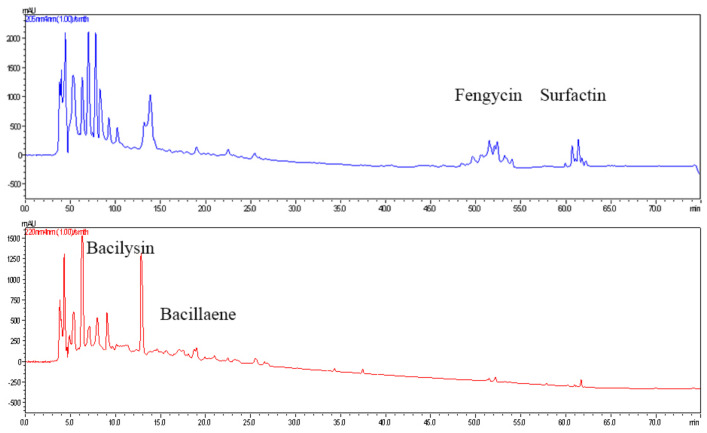
Ultra-performance liquid chromatography chromatograms of antifungal compounds produced by *Bacillus subtilis* L1-21.

The mass spectrometry data detected using MALDI-TOF-MS were analyzed and compared with the published research reported *Bacillus* metabolite and database Norine; we detected surfactin, fengycin, bacillaene and bacilysin in the Landy culture filtrate of *B. subtilis* L1-21 (Table 3; Figure 8). The sodiated molecules [M + Na]^+^ at *m*/*z* 1030.58, 1044.66, 1058.66, 1072.67 and 1086.71 were selected as precursor ions for further ESI-MS/MS analyses (Figures S2–S4). The ions at *m*/*z* 392 and 320 corresponded to ion formations [Leu–Leu–Asp + Na + CO]^+^ and [Leu–Leu–Val + Na–CO]^+^. The ions at *m*/*z* 707, 594, 481 and 463 matched ion formations [M + Na-SC-Glu]^+^, [M + Na-SC- Glu–Leu]^+^, [M + Na-SC- Glu–Leu–Leu]^+^ and [M + Na-SC- Glu–Leu –Leu–H_2_O]^+^, respectively. SC refers to the β-OH-fatty acid side chain. According to these regular ion formations, the amino acid sequence could be deduced as Glu–Leu–Leu–Val–Asp–Leu–Leu. Finally, the sodiated molecules [M + Na]^+^ at *m*/*z* 1030.58, 1044.66, 1058.66, 1072.67 and 1086.71 were identified as C_13_ surfactin A, C_14_ surfactin A, C_15_ surfactin A, C_16_ surfactin A and C_17_ surfactin A, respectively (Table 3; Figure S5). The protonated molecules [M + H]^+^ at *m*/*z* 1449.95, 1463.97, 1477.95, 1491.98 and 1505.99 were selected as precursor ions for further ESI-MS/MS analyses (Figure S6A–E). The ions at *m/z* 1080, 966 and 896 corresponding to ion formations [b_2_]^+^, [b_3_]^+^and [c_1_]^+^, respectively, were the characteristic fragment ions of fengycin A (Figure S6F). The ions at *m*/*z* 1108, 994 and 896 were the characteristic fragment ions of fengycin B (Figure S6F). In addition, the ions at *m*/*z* 1094, 980 and 896 corresponding to ion formations [b_2_]^+^, [b_3_]^+^and [c_1_]^+^, respectively, were the characteristic fragment ions of fengycin C (Figure S6F). The protonated molecules [M + H]^+^ at *m*/*z* 1449.95, 1463.97, 1477.95, 1491.98 and 1505.99 were deduced as C_15_ fengycin A, C_16_ fengycin A, C_16_ fengycin C, C_16_ fengycin B and C_17_ fengycin B, respectively (Table 3: Figure S6).

### 3.7. The Colonization Ability of Bacillus Subtilis L1-21

**Figure 9:**
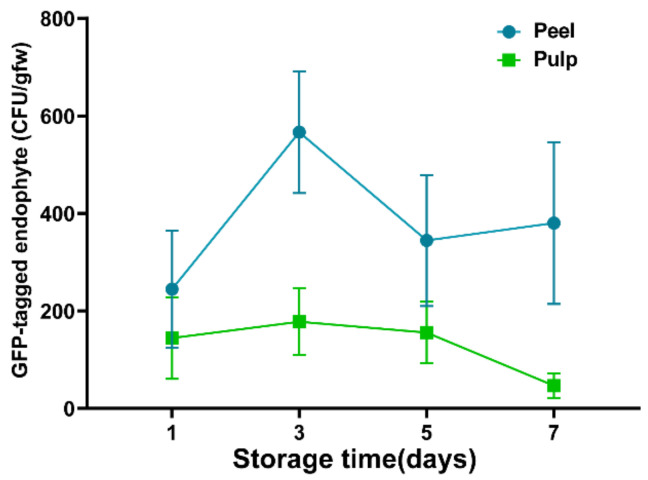
Colonization of *Bacillus subtilis* L1-21 on citrus fruits at different times. GFP-tagged endophyte L1-21 was used in this experiment to check the efficient colonization.

**Figure 10:**
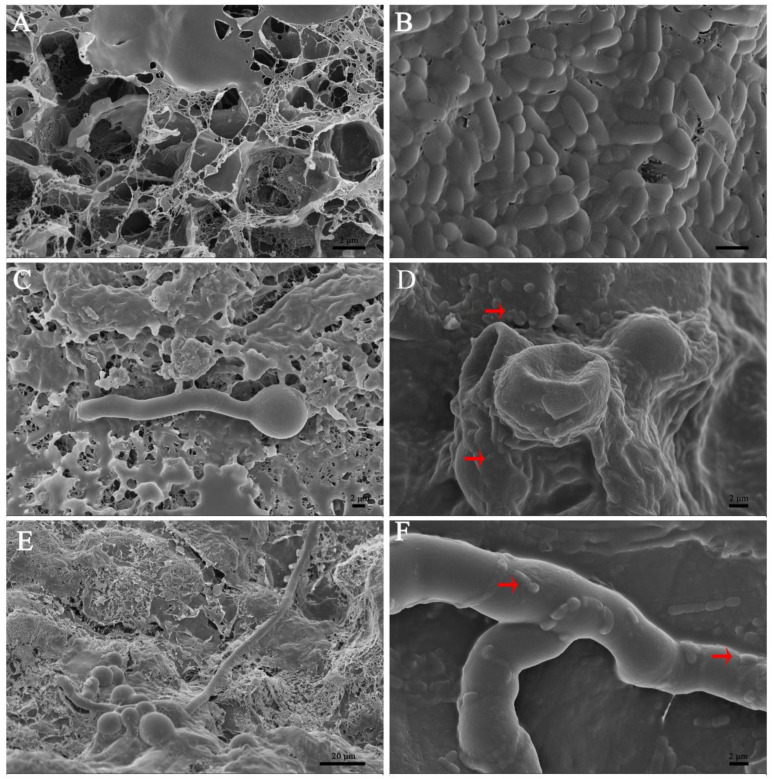
Scanning electron micrographs of colonization of endophyte *Bacillus subtilis* L1-21 on pathogens in citrus. Wound inoculation was carried out and the experiments were performed as (**A**) CK, 24h after treatment; (**B**) L1-21, 24 h after treatment; (**C**) *Penicillium digitatum* spore, 12 h after treatment, spores germinate normally; (**D**) *P. digitatum* spore + L1-21, 12 h after treatment, germinating spore deformity; (**E**) *P. digitatum* spores, 24 h after treatment; (**F**) *P. digitatum* spore + L1-21, 24 h after treatment.

Our results suggested that endophyte *B. subtilis* L1-21 could colonize in citrus fruit peel and pulp. The number of colonization was found to be highest after 3 d in both peel and pulp (Figure 9). The quantity of colonizers was higher in the peel than in the pulp. Our scanning electron microscopy results showed that *B. subtilis* L1-21 not only colonized inside the citrus fruit, but co-inoculation through wounds on pathogen *P. digitatum* mycelium showed that compared to the control (Figure 10A), it can easily colonize on the pathogen after 24 h, as occupying significant space (Figure 10B) further suppressed the pathogen spread. As shown in Figure 10C, the pathogen spores grow normally in the absence of *B. subtilis* L1-21, but the inoculation of this endophyte displayed marked deformation of pathogen spores after 24 h (Figure 10D). Our results also suggested that compared with pathogen control (Figure 10E), treatment with *B. subtilis* L1-21 helped the successful colonization on the pathogen spores (Figure 10F).

## 4. Discussion

The endophyte-mediated biocontrol of fungal pathogens has been studied extensively in recent decades due to the long-lasting effects on plants [25]. The bacterial strains in the *Bacillus* family are generally regarded as safe for plant protection and also used as probiotic consortia to reshape the microbial diversity of the host [31]. *Bacillus subtilis* L1-21 is an endophytic bacterium isolated from healthy citrus plants, with a wide range of colonization host and antagonistic effects against a variety of pathogenic fungi and bacteria [13,14]. In this study, *P. digitatum* was isolated and characterized as a causing agent of citrus green mold. Our results confirmed that *B. subtilis* L1-21 displayed a potentially antagonistic effect on *P. digitatum* that causes citrus green mold, a postharvest disease of citrus fruit.

In this respect, different biocontrol efficacies were assessed for numerous culture filtrates of *B. subtilis* L1-21 in in vivo experiments to provide resistance against pathogen *P. digitatum*. Fortunately, the biocontrol efficacy of the fermentation culture was demonstrated to be better for *B. subtilis* L1-21. Hence, this was similar to *B. subtilis* L1-21 against *Botrytis cinerea*, as reported by Bu [13]. The supernatant of *B. amyloliquefaciens* DH-4 was capable against *P. digitatum* [24]. They speculated that the biocontrol efficacy of DH-4 cell suspension was not as good as that of a supernatant, which may be because DH-4 could not grow well and produce enough antimicrobial substances on citrus peel [24]. Experiments confirmed that *B. subtilis* L1-21 could colonize the peel and pulp of citrus fruit. The highest colonization was evaluated on the third day, followed by an increase to the decrease trends of endophyte cells after 7 d. Therefore, we reasoned that the biocontrol efficacy of the cell suspension was better after 3 d, but it decreased in 7 d because none of the single colony was isolated on the culture medium.

In general, *Bacillus* is known to produce numerous antagonistic substances against postharvest pathogens [32]. Previous studies have reported that the production of antagonistic substances depends upon the suitable culture medium. In order to increase the content of antagonistic substances, the medium were screened for the maximum production of antagonistic substances from endophytic strain *B. subtilis* L1-21. We examined that the inhibition rate of Landy culture filtrate was better, which suggested that Landy medium is more suitable for the extraction of antifungal compounds from *B. subtilis* L1-21. The selection of key medium plays an important role in the production of desired lipopeptides [33]. Previous studies also suggested that Landy medium is suitable for the production of lipopeptides from the microbial strains [34]. Apart from the medium, temperature and shaking are the other important factors that need to be considered in the long run for the production of peptides [35]. The further use of the scanning electron microscope verified that a high concentration of the *B. subtilis* L1-21 Landy culture filter could inhibit spore germination and the spores began to deform after 36 h. Subsequently, a low concentration of the culture filtrate first deformed and expanded the pathogen spores, then the spores leaked, and finally, lost their vitality. In addition, a small part of the germinated spores could not normally develop into mycelium. These results provide a useful insight into the antagonistic mechanism of *B. subtilis* L1-21, which could deactivate the pathogen spores even at a lower concentration.

Further, a major hurdle in processing the potential bacterial strains for commercial purposes is the stability at a high temperature and pH value. We also analyzed the stability of *B. subtilis* L1-21 Landy medium’s sensitivity to UV light, 4–90 °C and different pH values, which showed similar results to previous reports [23]. However, the inhibition rate reduces significantly in the presence of 100 and 121 °C for 30 min, which is not conducive to the processing of a few fungicide formulations, as fungicide formulation processing uses a high temperature and does not last for 30 min. Hence, further investigation is required to evaluate the impact of higher temperature in a shorter time duration.

To gain further insights into the identification of antifungal compounds, we analyzed *B. subtilis* L1-21 Landy culture filter using LC-MS. The most featured compounds detected were surfactins, fengycins, bacillaene and bacilysin. Both surfactin and fengycin are lipopeptides that are reported to interfere with the cell membrane [36]. Li et al. [37] reported that surfactin, fengycin and bacillaene are responsible for inhibiting *P. digitatum*. In addition, Li et al. [37] also highlighted the antifungal activity of macroactin and iturin against *P. digitatum*, which were not detected in the Landy culture filtrate of *B. subtilis* L1-21. *Bacillus* sp. W176 was also found to produce macrolactin, bacillaene, mycosubtilin and surfactin against citrus green mold [23]. In this study, five surfactins and five fengycins were identified through MS/MS. However, it is not clear which monomer had the strongest antifungal activity against *P. digitatum*. Bacilysin (L-alanyl-[2,3-epoxycyclohexanone-4]-L- alanine) is a dipeptide antibiotic that shows antagonistic activity against a wide range of fungi and bacteria. Bacilysin not only inhibits fungi and bacteria, but also antagonizes cyanobacteria (*Microcystis aeruginosa*) [38]. However, its activity on *P. digitatum* has not been reported before.

In this work, we identified 13 compounds produced by *B. subtilis* L1-21 using GC-MS. Among them, phenol attained significant antifungal activity, also reported against *Fusarium oxysporum* f. sp. *cubense,* produced by *B. amyloliquefaciens* NJN-6 [39]. However, dodecane produced by *B. amyloliquefaciens* NJN-6 exhibited antifungal activity against *F. oxysporum* f. sp. *cubense*. Phenylethyl alcohol showed antifungal activity against *Fragaria ananassa* cv. Maehyang and prolonged the postharvest life of strawberries [40]. *Salmonella Typhi* pathogen was successfully inhibited with hexadecanoic acid [41,42] whereas benzeneacetic acid demonstrated potential antagonistic activity against a variety of bacteria and fungi [43,44]. However, the contents of these compounds were very low, and their antifungal activity requires further verification. The substance with a retention time of 6.919 was not identified (Figure S1), and its relative content was 32.28%. In summary, our results suggested that *B. subtilis* L1-21 can be cultured in Landy medium to control citrus green mold. Future work will focus on optimizing a cheaper medium that can promote a higher production of antagonistic substances and a mode of action of these components against pathogens.

## 5. Conclusions

In conclusion, we suggest that *B. subtilis* L1-21, previously used against the pathogen causing citrus huanglongbing, can play a prominent role to reduce the pathogen *P. digitatum* during fruit storage. *Bacillus subtilis* L1-21 produces important antifungal compounds in the form of surfactins, fengycins, bacillaene and bacilysin to mitigate the pathogen. We also highlighted that a low concentration of the *B. subtilis* L1-21 Landy culture filtrate was able to deform and expand the pathogen spores, which resulted in a loss of vitality. The endophyte can easily colonize in peel and pulp, providing no room for the pathogen to invade easily. The potential endophyte can serve as an important bioagent to reduce the incidence of pathogens through citrus–endophyte pathogen interaction on other kinds of citrus fruits present in the food industry. Further characterization of the molecular mechanism of *B. subtilis* L1-21 to control this pathogen will provide an exciting venue for future research.

## Figures and Tables

**Table 1:** Components of ethyl acetate extract from *Bacillus subtilis* L1-21 by using GC-MS analysis.

No	Retention Index	Compound	Exact Mass	Formula	Q ^a^ (%)	Relative Content(%) ^b^
1	6.387	2-Butenoic acid, 3-methyl-	100.05	C_5_H_8_O_2_	97	0.21
2	7.125	Phenol	94.04	C_6_H_6_O	91	0.43
3	9.185	Phenylethyl Alcohol	122.07	C_8_H_10_O	87	0.15
4	10.455	Dodecane	170.20	C_12_H_26_	97	0.14
5	10.873	Benzofuran, 2,3-dihydro-	120.06	C_8_H_8_O	90	0.2
6	11.514	Benzeneacetic acid	136.05	C_8_H_8_O_2_	94	0.53
7	15.388	Tetradecane	198.24	C_14_H_30_	98	1.15
8	20.400	Hexadecane	226.27	C_16_H_34_	98	0.49
9	22.689	Z-11-Tetradecenoic acid	226.19	C_14_H_26_O_2_	91	0.21
10	23.645 25.676 25.756	Pyrrolo[1,2-a]pyrazine-1,4-dione, hexahydro-3-(phenylmethyl)-	244.12	C_14_H_16_N_2_O_2_	94 95 99	2.80 6.60 3.67
11	24.646	1,2-Benzenedicarboxylic acid, bis (2-methylpropyl) ester	278.15	C_16_H_22_O_4_	91	3.62
12	26.071	n-Hexadecanoic acid	256.24	C_16_H_32_O_2_	98	0.63
13	28.617	Octadecanoic acid	284.27	C_18_H_36_O_2_	98	0.33

^a^ Q for match quality. ^b^ peak area relative to the total peak area.

**Table 2:** Effect of *Bacillus subtilis* L1-21 culture filtrate on spore germination of pathogen.

Culture Medium Type	Inhibition Rate (%)			
	Culture Time (h)			
	24	48	72	96
NB	93.98 ± 1.34 a	95.39 ± 1.31 ab	94.02 ± 0.94 a	91.38 ± 0.91 c ^a^
Landy	95.61 ± 0.85 a	94.62 ± 0.48 ab	98.32 ± 0.33 a	96.57 ± 0.30 ab
TSA	6.00 ± 1.53 c	2.25 ± 1.68 d	5.22 ± 0.94 d	4.26 ± 0.96 e
10% TSA	38.86 ± 7.13 b	66.70 ± 3.32 c	36.32 ± 5.88 c	48.09 ± 3.35 d
R_2_A	38.92 ± 6.35 b	90.09 ± 1.24 b	87.16 ± 1.08 a	92.57 ± 2.53 bc
MT	94.92 ± 1.21 a	93.74 ± 0.37 ab	76.15 ± 4.94 b	87.66 ± 0.84 c
Prochloraz (Positive Control)	98.34 ± 0.55 a	98.34 ± 0.55 a	98.34 ± 0.55 a	98.34 ± 0.55 a

^a^ Different letters indicate the significant differences (*p* < 0.05).

**Table 3:** Antifungal components of Landy culture filtrate from *Bacillus subtilis* L1-21 by using LC-MS analysis.

Metabolite	Retention Index	Mass Peak, *m*/*z*		Characteristic Fragment Ions	MW ^a^	Assignment
	(min)	[M+H]^+^	[M+Na]^+^			
Bacilysin	7.652	-	293.71	-	270.2	Bacilysin
Bacillaene	17.567	581.78	-	-	580.4	Bacillaene
Fengycin	52.680	1449.95	-	1080, 966, 896	1448.8	C_15_ fengycinA
	50.638–51.103	1463.97	-	1080, 966, 896	1462.8	C_16_ fengycinA
	51.257	1477.95	-	1094, 980, 896	1477.8	C_16_ fengycinC
	51.407	1491.98	-	1108, 994, 896	1491.8	C_16_fengycinB
	52.377	1505.99	-	1108, 994, 896	1504.8	C_17_ fengycinB
Surfactin	60.078–60.490	-	1030.58	707, 594, 481	1007.7	C_13_ surfactinA
	60.933–62.042	-	1044.66	707, 594, 481	1021.7	C_14_ surfactinA
	61.503–62.200	-	1058.66	707, 594, 481	1035.7	C_15_ surfactinA
	61.978–62.453	-	1072.67	707, 594, 481	1049.7	C_16_ surfactinA
	63.087	-	1086.71	707, 594, 481	1063.7	C_17_ surfactinA

^a^ MW = molecular weight.

## Data Availability

All the data are present inside manuscript file.

## Supplementary Materials

The following are available online at https://www.mdpi.com/article/10.3390/jof8010080/s1. Figure S1. GC-MS analysis of compounds from *Bacillus subtilis* L1-21, Figure S2. LC-MS (A) Baciysin,[M+Na]^+^293.17, (B) Bacillaene,[M+H]^+^581.78.Figure S3. LC-MS fengycin A[M+H]^+^1449.95, B[M+H]^+^1463.97, C[M+H]^+^1477.95, D[M+H]^+^1491.98, E[M+H]^+^1505.99. Figure S4. LC-MS Surfactin A[M+Na]^+^1030.58, B[M+Na]^+^1044.66, C[M+Na]^+^1058.66, D[M+Na]^+^1072.67, E[M+Na]^+^1086.71. Figure S5. MS/MS Surfactin A [M+Na]^+^1030.58, C_13_ surfactin A, B[M+Na]^+^1044.66, C_14_ surfactin A, C[M+Na]^+^1058.66, C_15_ surfactin A, D[M+Na]^+^1072.67, C_16_ surfactin A, E[M+Na]^+^1086.71, C_17_ surfactin A, F The structure of Surfactin A. Figure S6. MS/MS fengycin A[M+H]^+^1449.95, C_15_ fengycin A, B[M+H]^+^1463.97, C_16_ fengycin A, C[M+H]^+^1477.95, C_16_ fengycin C, D[M+H]^+^1491.98, C_16_ fengycin B, E[M+H]^+^1505.99, C_17_ fengycin B, F The structure of fengycin A, fengycin, B fengycin C.
