# Supplementary material for: Developing Penicillium digitatum Management Strategies on Post-Harvest Citrus Fruits with Metabolic Components and Colonization of Bacillus subtilis L1-21
Source: J Fungi (Basel). 2022 Jan 14;8(1):80. doi: 10.3390/jof8010080 (PMC8777893; doi:10.3390/jof8010080)
Supplement: Supplementary file 1 [file jof-08-00080-s001.zip › jof-1544088-supplementary.pdf]

Journal of
Fungi

MDPI

Supplementary materials

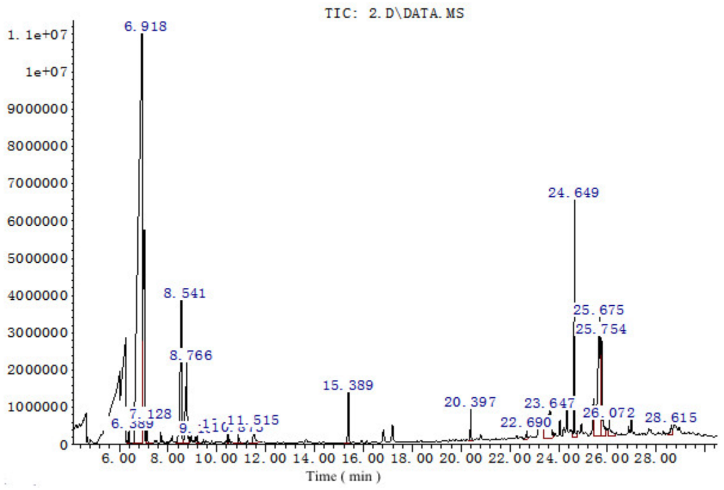

Figure S1. GC-MS analysis of compounds from *Bacillus subtilis* L1-21.

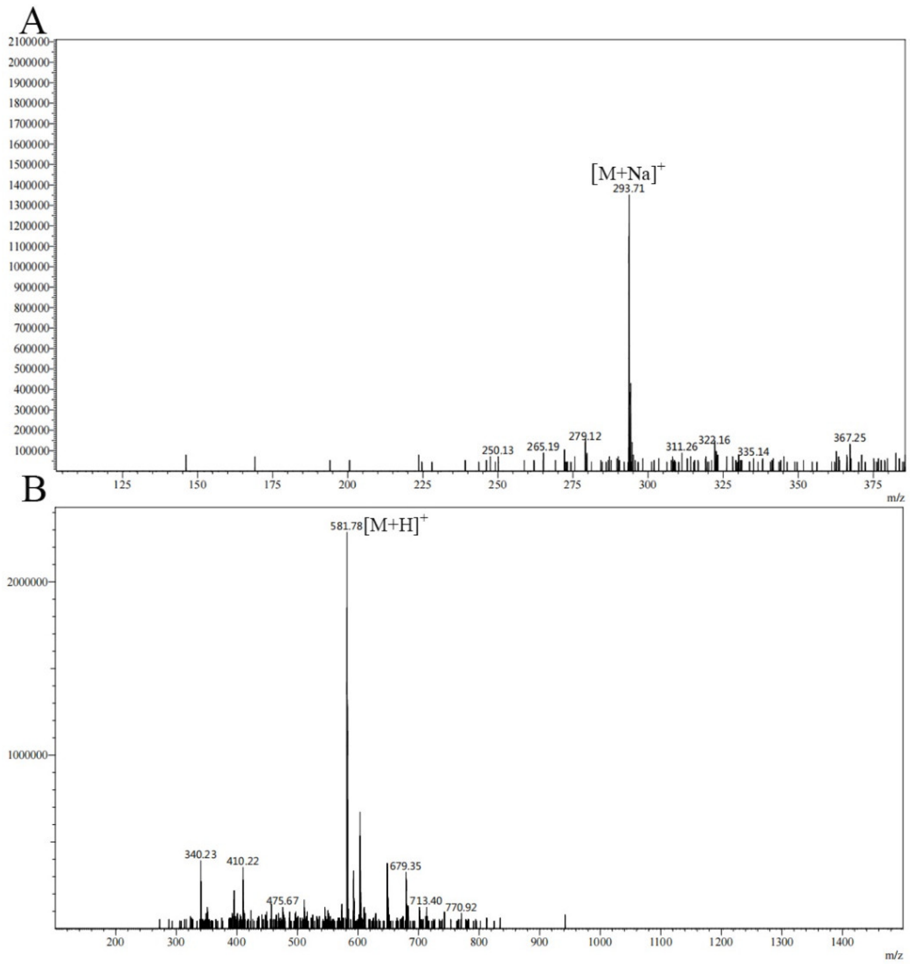

Figure S2. LC-MS (**A**) Baciysin,[M+Na]+293.17, (**B**) Bacillaene,[M+H]+581.78.

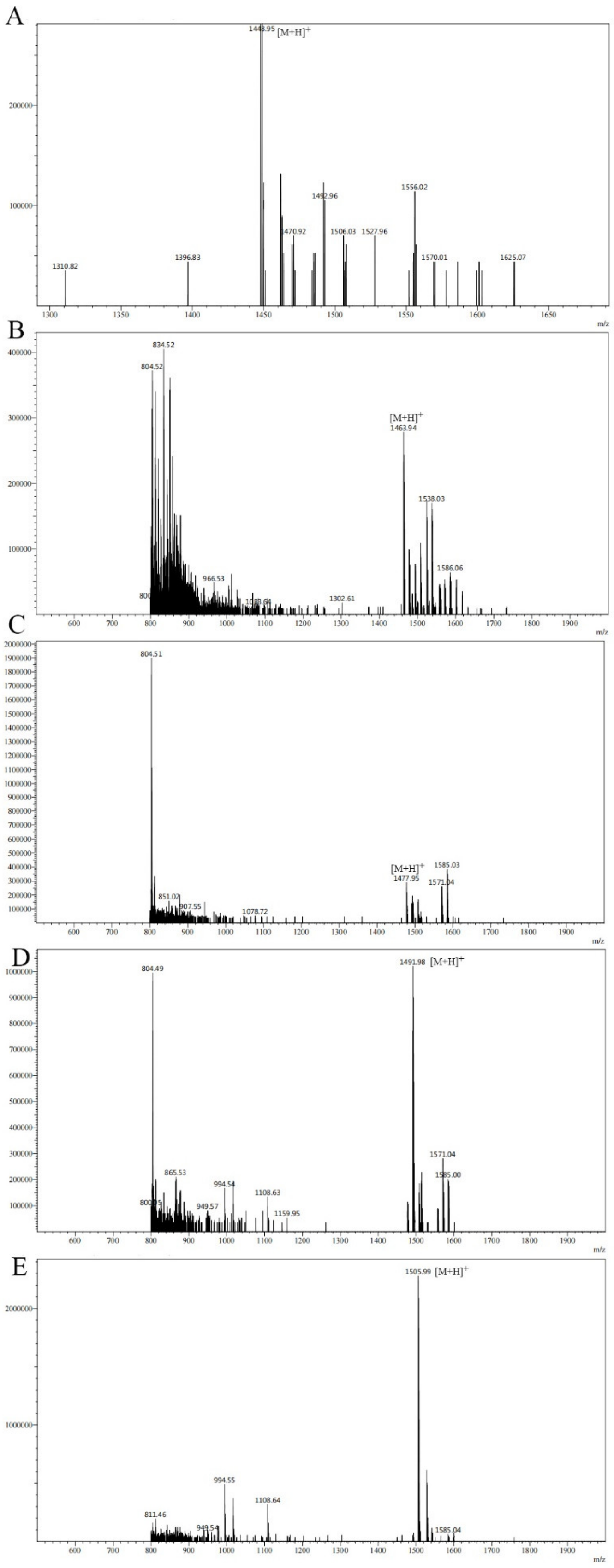

Figure S3. LC-MS fengycin A[M+H]+1449.95, B[M+H]+1463.97, C[M+H]+1477.95, D[M+H]+1491.98, E[M+H]+1505.99.

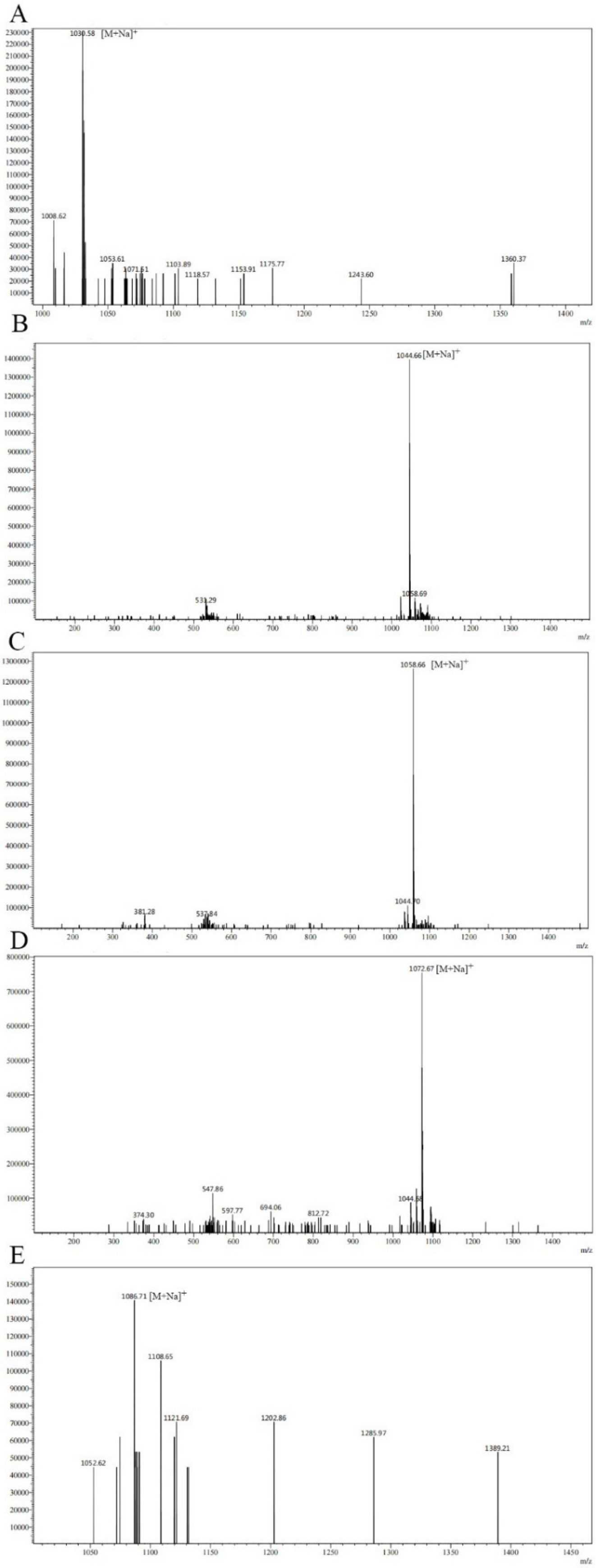

Figure S4. LC-MS Surfactin A[M+Na]+1030.58, B[M+Na]+1044.66, C[M+Na]+1058.66.D[M+Na]+1072.67,E[M+Na]+1086.71.

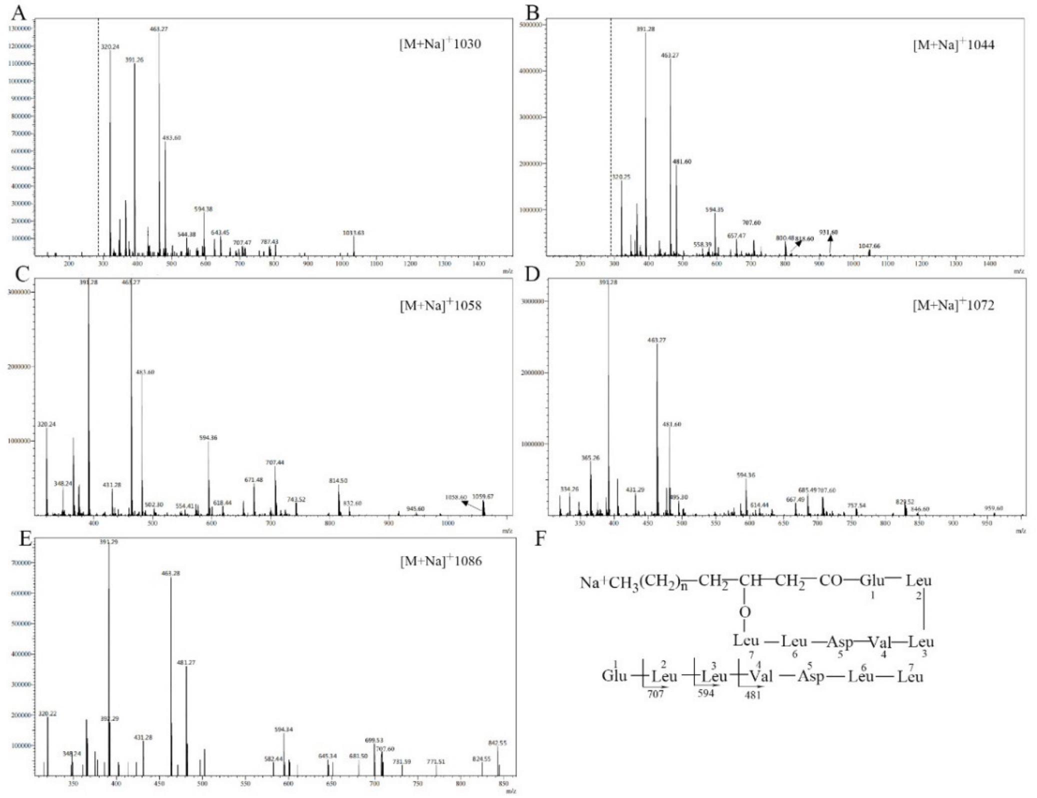

Figure S5. MS/MS Surfactin A [M+Na]+1030.58,C_{13} surfactin A, B[M+Na]+1044.66, C_{14} surfactin A, C[M+Na]+1058.66, C_{15} surfactin A, D[M+Na]+1072.67,C_{16} surfactin A,.E[M+Na]+1086.71 C_{17} surfactin A, F The structure of Surfactin A.

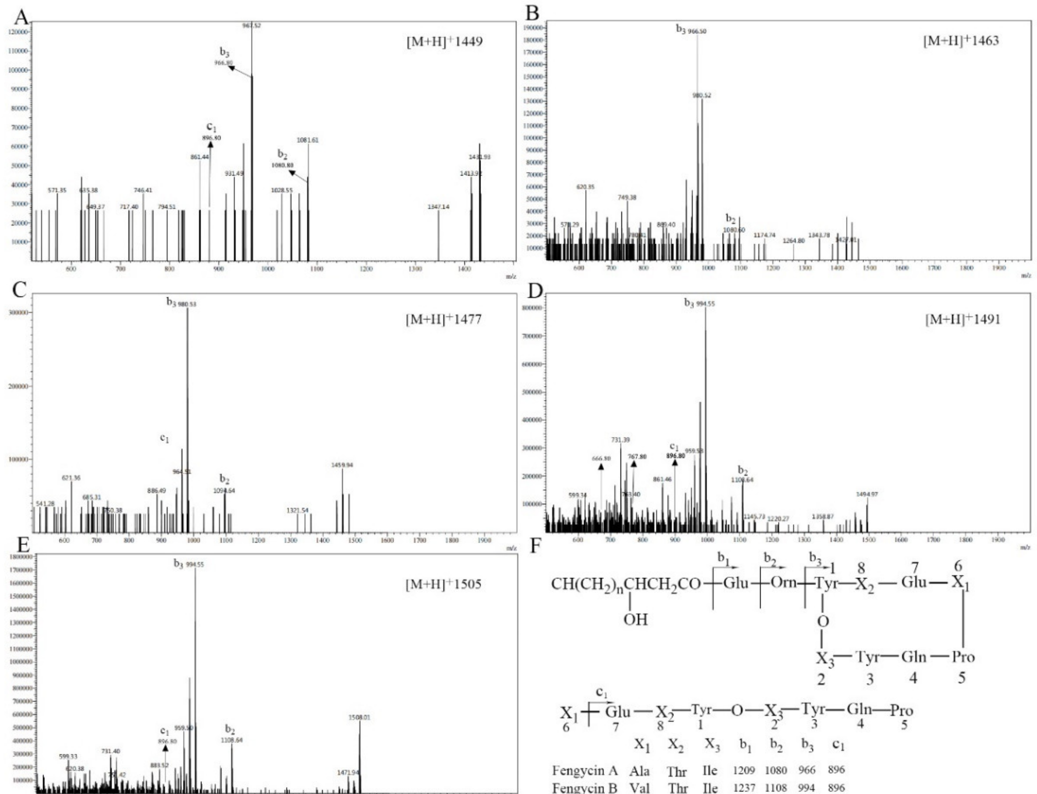

Figure S6. MS/MS fengycin A[M+H]+1449.95, C_{15} fengycin A, B[M+H]+1463.97, C_{16} fengycin A, C[M+H]+1477.95, C_{16} fengycin C, D[M+H]+1491.98, C_{16} fengycin B , E[M+H]+1505.99,C_{17} fengycin B, F The structure of fengycin A, fengycin, B fengycin C.
